# 2,2-Dichloro-3,7,7,11-tetra­methyl-10-aza­tetra­cyclo­[6.5.0.0^1,3^.0^9,11^]trideca­ne

**DOI:** 10.1107/S1600536813007642

**Published:** 2013-03-28

**Authors:** Abdelouahd Oukhrib, Ahmed Benharref, Mohamed Saadi, Moha Berraho, Lahcen El Ammari

**Affiliations:** a Laboratoire de Chimie Biomoléculaire, Substances Naturelles et, Réactivité "Unité Associée au CNRST (URAC16)", Université Cadi Ayyad, Faculté des Sciences Semlalia, BP 2390, Bd My Abdellah, 40000 Marrakech, Morocco; b Laboratoire de Chimie du Solide Appliquée, Université Mohammed V-Agdal, Faculté des Sciences, Avenue Ibn Battouta, BP 1014, Rabat, Morocco

## Abstract

The title compound, C_16_H_25_Cl_2_N, was synthesized from β-himachalene (3,5,5,9-tetra­methyl-2,4a,5,6,7,8-hexa­hydro-1*H*-benzocyclo­heptene), which was isolated from the essential oil of the Atlas cedar (*Cedrus Atlantica*). The mol­ecule is built up from fused six- and seven-membered rings linked to two three-membered rings. The six-membered ring shows a half-chair conformation, whereas the seven-membered ring displays a boat conformation. The dihedral angle between the mean planes through the six- and seven-membered rings is 59.8 (2)°. The two three-membered rings lie to one side and each is nearly perpendicular to the six-membered ring, forming dihedral angles of 83.2 (2) and 86.0 (2)°. The absolute structure was established unambiguously from anomalous dispersion effects. No specific inter­molecular inter­actions are noted in the crystal structure.

## Related literature

For the isolation of β-himachalene, see: Joseph & Dev (1968 ▶); Plattier & Teisseire (1974 ▶). For the reactivity of this sesquiterpene, see: Lassaba *et al.* (1998 ▶); Chekroun *et al.* (2000 ▶); El Jamili *et al.* (2002 ▶); Sbai *et al.* (2002 ▶); Dakir *et al.* (2004 ▶). For its biological activity, see: Daoubi *et al.* (2004 ▶). For a similar compound, see: Benharref *et al.* (2010 ▶). For puckering calculations, see: Cremer & Pople (1975 ▶). For the Flack parameter refinement, see: Flack & Bernardinelli (2000 ▶).
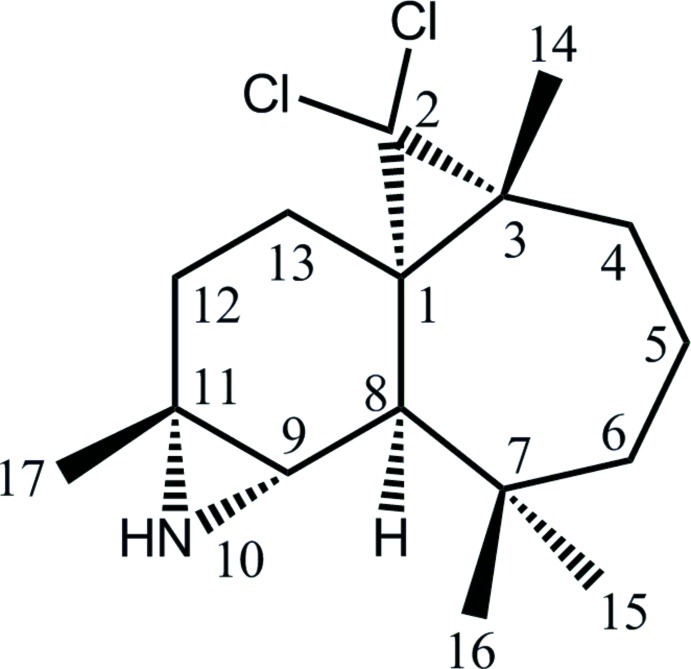


## Experimental

### 

#### Crystal data

C_16_H_25_Cl_2_N
*M* *_r_* = 302.27
Orthorhombic, 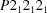

*a* = 8.607 (3) Å
*b* = 13.222 (4) Å
*c* = 13.973 (4) Å
*V* = 1590.2 (8) Å^3^
*Z* = 4
Mo *K*α radiation
μ = 0.40 mm^−1^
*T* = 296 K
0.43 × 0.31 × 0.28 mm

#### Data collection

Bruker X8 APEX Diffractometer
22014 measured reflections
3261 independent reflections
2924 reflections with *I* > 2σ(*I*)
*R*_int_ = 0.048

#### Refinement

*R*[*F* ^2^ > 2σ(*F* ^2^)] = 0.049
*wR*(*F* ^2^) = 0.144
*S* = 1.06
3261 reflections
172 parameters
H-atom parameters constrained
Δρ_max_ = 0.33 e Å^−3^
Δρ_min_ = −0.37 e Å^−3^
Absolute structure: Flack & Bernardinelli (2000 ▶), 1385 Friedel pairs
Flack parameter: 0.12 (9)

### 

Data collection: *APEX2* (Bruker, 2009 ▶); cell refinement: *SAINT* (Bruker, 2009 ▶); data reduction: *SAINT*; program(s) used to solve structure: *SHELXS97* (Sheldrick, 2008 ▶); program(s) used to refine structure: *SHELXL97* (Sheldrick, 2008 ▶); molecular graphics: *ORTEP-3 for Windows* (Farrugia, 2012 ▶); software used to prepare material for publication: *PLATON* (Spek, 2009 ▶) and *publCIF* (Westrip, 2010 ▶).

## Supplementary Material

Crystal structure: contains datablock(s) I, global. DOI: 10.1107/S1600536813007642/tk5210sup1.cif

Structure factors: contains datablock(s) I. DOI: 10.1107/S1600536813007642/tk5210Isup2.hkl

Supplementary material file. DOI: 10.1107/S1600536813007642/tk5210Isup3.cml

Additional supplementary materials:  crystallographic information; 3D view; checkCIF report

## Figures and Tables

**Fig. 1.**
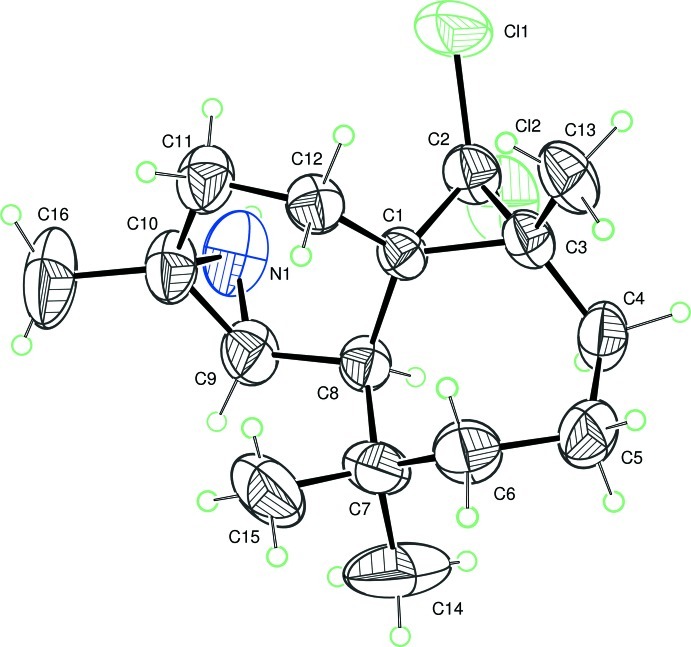
Molecular structure of the title compound with the atom-labelling scheme. Displacement ellipsoids are drawn at the 50% probability level. H atoms are represented as small spheres of arbitrary radii.

## supplementary crystallographic information

### Comment

Our work lies within the framework of "value-adding" to the most abundant essential oils in Morocco, such as *Cedrus atlantica*. This oil is made up mainly (75%) of bicyclic sesquiterpenes hydrocarbons, among which is found the compound, β-himachalene (Joseph & Dev, 1968; Plattier & Teisseire, 1974). The reactivity of this sesquiterpene and its derivatives has been studied extensively by our team in order to prepare new products having biological properties (Lassaba *et al.*, 1998; Chekroun *et al.*, 2000; El Jamili *et al.*, 2002; Sbai *et al.*, 2002; Dakir *et al.*, 2004). Indeed, these compounds were tested, using the food poisoning technique, for their potential anti-fungal activity against phytopathogen *Botrytis cinerea* (Daoubi *et al.*, 2004). Thus the action of one equivalent of dichlorocarbene, generated *in situ* from chloroform in the presence of sodium hydroxide as base and *n*-benzyltriethylammonium chloride as catalyst, on β-himachalene produces only (1*S*,3*R*,8*R*)-2,2-dichloro-3,7,7,10-tetramethyltricyclo [6.4.0.0^1,3^] dodec-9-ene (El Jamili *et al.*, 2002). Treatment of the latter with one equivalent of *meta*-chloroperbenzoic acid (mCPBA) leads (1*S*,3*R*,8*S*,9*R*,10*S*)-2,2-dichloro-9β–10β-epoxy-3,7,7,10-tetramethyl-tricyclo[6.4.0.0^1,3^]dodecane (Benharref *et al.*, 2010). Treating said epoxide with sodium azide in the presence of ammonium chloride followed by Ph_3_P led to 2,2- dichloro-3,7,7,11-tetramethyl-10- aza-tetracyclo [6,5,0,0^1.3^,0^9.11^]tridecane with a yield of 82.5% from azido alcohol. The structure of this new product was determined by X-ray diffraction analysis.

The molecule contains a fused six- and seven-membered rings, which is fused to two three-membered rings as shown in Fig.1. The six-membered ring has a half chair conformation as indicated by the total puckering amplitude QT = 0.461 (3) Å and spherical polar angle θ = 133.2 (4)° and φ2 = 161.2 (5)°, whereas the seven-membered ring displays a boat conformation with QT = 1.157 (3) Å, θ2 = 88.24 (15)°, φ2 = -47.69 (15)° and φ3 = -106 (4)° (Cremer & Pople, 1975). The dihedral angle between the six and seven-membered rings is 59.8 (2)°. The three-membered rings (C1C2C3) and (C9N1C10) are nearly perpendicular to the six-membered ring (C1C8C9C11C12C13) with a dihedral angle of 86.0 (2) and 83.2 (2)°, respectively. Owing to the presence of Cl atoms, the absolute configuration could be fully confirmed from anomalous dispersion effects, by refining the Flack parameter as C1(*S*), C3(*R*), C8(*R*), C9(*S*), and C10(*R*).

### Experimental

A mixture of epoxide, (1*S*,3*R*,8*S*,9*R*,10*S*)-2,2-dichloro-9β–10β-epoxy- 3,7,7, 10-tetramethyl-tricyclo[6.4.0.0^1,3^] dodecane (1.5 g, 5 mmol), NaN_3_ (3.4 g, 53.24 mmol), and NH_4_Cl (0.57 g, 10.65 mmol) in MeOH (30 ml) and water (2.5 ml) was heated to reflux two hours. The reaction mixture was cooled to room temperature, diluted with water (40 ml) and extracted with ethyle acetate (3 *x* 30 ml). The combined organic layers were washed with brine, dried (MgSO_4_), concentrated, and chromatographed (15% EtOAc in hexane) to provide 1.15 g (80%) of azido alcohol. To a solution of this azido alcohol (0.7 g, 2 mmol) in dry acetonitrile (20 ml) was added Ph_3_P (0.95 g, 3.6 mmol) and the reaction was heated to reflux for 1.5 h. The reaction mixture was cooled to room temperature, concentrated and chromatographed (4%MeOH in CH_2_Cl_2_) to provide 0.5 g (1.65 mmol) of 2,2- dichloro-3,7,7,11- tetramethyl-10-aza-tetracyclo[6,5,0,0^1.3^,*O*^9.11^]tridecane with a yield of 82.5% from azido alcohol. The title compound was recrystallized from its ethyl acetate solution.

### Refinement

All H atoms were fixed geometrically and treated as riding with N—H = 0.86 Å, C—H = 0.96 Å (methyl), 0.97 Å (methylene), 0.98 Å (methine) with *U*_iso_(H) = 1.2 *U*_eq_(amine, methylene, methine) or *U*_iso_(H) = 1.5 *U*_eq_(methyl). The 1385 Friedel opposites reflections are not merged. Owing to poor agreement, one reflection, *i.e*. (0 1 1), was removed from the final cycles of refinement.

### Crystal data

**Table:**

C_16_H_25_Cl_2_N	*F*(000) = 648
*M**_r_* = 302.27	*D*_x_ = 1.263 Mg m^−^^3^
Orthorhombic, *P*2_1_2_1_2_1_	Mo *K*α radiation, λ = 0.71073 Å
Hall symbol: P 2ac 2ab	Cell parameters from 3261 reflections
*a* = 8.607 (3) Å	θ = 2.8–26.4°
*b* = 13.222 (4) Å	µ = 0.40 mm^−^^1^
*c* = 13.973 (4) Å	*T* = 296 K
*V* = 1590.2 (8) Å^3^	Block, colourless
*Z* = 4	0.43 × 0.31 × 0.28 mm

### Data collection

**Table:**

Bruker X8 APEX Diffractometer	2924 reflections with *I* > 2σ(*I*)
Radiation source: fine-focus sealed tube	*R*_int_ = 0.048
Graphite monochromator	θ_max_ = 26.4°, θ_min_ = 2.8°
φ and ω scans	*h* = −10→10
22014 measured reflections	*k* = −16→16
3261 independent reflections	*l* = −17→15

### Refinement

**Table:**

Refinement on *F*^2^	Secondary atom site location: difference Fourier map
Least-squares matrix: full	Hydrogen site location: inferred from neighbouring sites
*R*[*F*^2^ > 2σ(*F*^2^)] = 0.049	H-atom parameters constrained
*wR*(*F*^2^) = 0.144	*w* = 1/[σ^2^(*F*_o_^2^) + (0.0883*P*)^2^ + 0.3114*P*] where *P* = (*F*_o_^2^ + 2*F*_c_^2^)/3
*S* = 1.06	(Δ/σ)_max_ = 0.001
3261 reflections	Δρ_max_ = 0.33 e Å^−^^3^
172 parameters	Δρ_min_ = −0.37 e Å^−^^3^
0 restraints	Absolute structure: Flack & Bernardinelli (2000), 1385 Friedel pairs
Primary atom site location: structure-invariant direct methods	Flack parameter: 0.12 (9)

### Special details

**Table:**

Geometry. All e.s.d.'s (except the e.s.d. in the dihedral angle between two l.s. planes) are estimated using the full covariance matrix. The cell e.s.d.'s are taken into account individually in the estimation of e.s.d.'s in distances, angles and torsion angles; correlations between e.s.d.'s in cell parameters are only used when they are defined by crystal symmetry. An approximate (isotropic) treatment of cell e.s.d.'s is used for estimating e.s.d.'s involving l.s. planes.
Refinement. Refinement of *F*^2^ against all reflections. The weighted *R*-factor *wR* and goodness of fit *S* are based on *F*^2^, conventional *R*-factors *R* are based on *F*, with *F* set to zero for negative *F*^2^. The threshold expression of *F*^2^ > σ(*F*^2^) is used only for calculating *R*-factors(gt) *etc*. and is not relevant to the choice of reflections for refinement. *R*-factors based on *F*^2^ are statistically about twice as large as those based on *F*, and *R*- factors based on all data will be even larger.

### Fractional atomic coordinates and isotropic or equivalent isotropic displacement parameters (Å^2^)

**Table:**

	*x*	*y*	*z*	*U*_iso_*/*U*_eq_	
C1	0.4201 (3)	0.11081 (16)	0.12828 (15)	0.0369 (4)	
C2	0.5296 (3)	0.16233 (19)	0.1977 (2)	0.0484 (6)	
C3	0.3732 (3)	0.20831 (17)	0.18198 (19)	0.0434 (5)	
C4	0.2502 (3)	0.1978 (2)	0.2585 (2)	0.0565 (7)	
H4A	0.2472	0.2589	0.2968	0.068*	
H4B	0.2763	0.1418	0.3002	0.068*	
C5	0.0892 (4)	0.1794 (3)	0.2136 (3)	0.0702 (9)	
H5A	0.0222	0.1493	0.2616	0.084*	
H5B	0.0446	0.2442	0.1962	0.084*	
C6	0.0895 (3)	0.1110 (3)	0.1247 (2)	0.0659 (8)	
H6A	−0.0176	0.1018	0.1047	0.079*	
H6B	0.1420	0.1472	0.0737	0.079*	
C7	0.1632 (3)	0.0070 (2)	0.1324 (2)	0.0580 (7)	
C8	0.3404 (3)	0.01312 (17)	0.15959 (18)	0.0417 (5)	
H8	0.3455	0.0116	0.2296	0.050*	
C9	0.4322 (4)	−0.07729 (18)	0.1245 (2)	0.0571 (7)	
H9	0.3835	−0.1432	0.1356	0.069*	
C10	0.5372 (4)	−0.0736 (2)	0.0465 (2)	0.0587 (7)	
C11	0.5738 (4)	0.0256 (2)	−0.0032 (2)	0.0586 (7)	
H11A	0.5704	0.0151	−0.0718	0.070*	
H11B	0.6788	0.0458	0.0134	0.070*	
C12	0.4628 (3)	0.11134 (17)	0.02269 (18)	0.0463 (5)	
H12A	0.5110	0.1755	0.0069	0.056*	
H12B	0.3689	0.1050	−0.0152	0.056*	
C13	0.3622 (4)	0.30807 (19)	0.1274 (2)	0.0622 (8)	
H13A	0.2553	0.3283	0.1229	0.093*	
H13B	0.4041	0.2995	0.0643	0.093*	
H13C	0.4201	0.3592	0.1607	0.093*	
C14	0.0838 (5)	−0.0597 (4)	0.2083 (4)	0.1120 (17)	
H14A	0.1340	−0.1245	0.2105	0.168*	
H14B	−0.0237	−0.0685	0.1919	0.168*	
H14C	0.0915	−0.0277	0.2698	0.168*	
C15	0.1378 (5)	−0.0448 (3)	0.0346 (3)	0.0918 (13)	
H15A	0.1827	−0.1113	0.0358	0.138*	
H15B	0.1865	−0.0055	−0.0148	0.138*	
H15C	0.0285	−0.0499	0.0219	0.138*	
C16	0.5914 (7)	−0.1679 (2)	−0.0021 (3)	0.0979 (15)	
H16A	0.6623	−0.1507	−0.0526	0.147*	
H16B	0.5037	−0.2031	−0.0285	0.147*	
H16C	0.6429	−0.2106	0.0435	0.147*	
N1	0.5979 (4)	−0.0721 (2)	0.1464 (2)	0.0727 (8)	
H1	0.6735	−0.0698	0.1866	0.087*	
Cl1	0.69426 (9)	0.22566 (7)	0.15164 (8)	0.0806 (3)	
Cl2	0.57859 (12)	0.10793 (7)	0.30962 (6)	0.0787 (3)	

### Atomic displacement parameters (Å^2^)

**Table:**

	*U*^11^	*U*^22^	*U*^33^	*U*^12^	*U*^13^	*U*^23^
C1	0.0393 (10)	0.0313 (9)	0.0401 (11)	0.0019 (9)	−0.0026 (9)	0.0007 (8)
C2	0.0426 (13)	0.0495 (13)	0.0530 (14)	0.0034 (10)	−0.0124 (11)	−0.0055 (12)
C3	0.0428 (12)	0.0363 (11)	0.0511 (13)	0.0038 (9)	−0.0107 (10)	−0.0061 (10)
C4	0.0541 (15)	0.0578 (15)	0.0577 (16)	0.0119 (13)	−0.0002 (13)	−0.0190 (12)
C5	0.0458 (15)	0.0760 (19)	0.089 (2)	0.0104 (14)	0.0044 (16)	−0.0241 (17)
C6	0.0408 (13)	0.0736 (18)	0.083 (2)	0.0009 (13)	−0.0108 (14)	−0.0123 (16)
C7	0.0523 (16)	0.0500 (14)	0.0716 (19)	−0.0133 (12)	−0.0001 (14)	−0.0002 (13)
C8	0.0499 (13)	0.0351 (10)	0.0402 (11)	0.0013 (9)	0.0042 (10)	0.0026 (9)
C9	0.0725 (18)	0.0337 (11)	0.0652 (16)	0.0058 (11)	0.0107 (15)	0.0050 (11)
C10	0.081 (2)	0.0435 (13)	0.0513 (14)	0.0133 (13)	0.0098 (14)	−0.0016 (11)
C11	0.0768 (19)	0.0514 (13)	0.0476 (14)	0.0046 (13)	0.0133 (15)	0.0026 (12)
C12	0.0583 (14)	0.0389 (10)	0.0417 (12)	−0.0006 (11)	0.0012 (11)	0.0066 (9)
C13	0.0754 (19)	0.0315 (11)	0.080 (2)	0.0063 (12)	−0.0126 (16)	−0.0043 (12)
C14	0.073 (3)	0.111 (3)	0.152 (4)	−0.030 (2)	0.025 (3)	0.043 (3)
C15	0.086 (3)	0.076 (2)	0.113 (3)	−0.021 (2)	−0.016 (2)	−0.032 (2)
C16	0.158 (4)	0.0552 (17)	0.080 (2)	0.038 (2)	0.038 (3)	−0.0021 (16)
N1	0.0837 (19)	0.0743 (16)	0.0603 (14)	0.0316 (14)	−0.0066 (14)	0.0046 (13)
Cl1	0.0445 (4)	0.0787 (5)	0.1185 (8)	−0.0163 (3)	−0.0029 (4)	−0.0138 (5)
Cl2	0.0834 (6)	0.0917 (6)	0.0611 (4)	0.0245 (5)	−0.0341 (4)	−0.0037 (4)

### Geometric parameters (Å, º)

**Table:**

C1—C2	1.514 (3)	C9—N1	1.460 (5)
C1—C12	1.521 (3)	C9—H9	0.9800
C1—C8	1.527 (3)	C10—C16	1.495 (4)
C1—C3	1.545 (3)	C10—N1	1.491 (4)
C2—C3	1.493 (3)	C10—C11	1.517 (4)
C2—Cl1	1.767 (3)	C11—C12	1.526 (4)
C2—Cl2	1.772 (3)	C11—H11A	0.9700
C3—C4	1.511 (4)	C11—H11B	0.9700
C3—C13	1.527 (4)	C12—H12A	0.9700
C4—C5	1.540 (4)	C12—H12B	0.9700
C4—H4A	0.9700	C13—H13A	0.9600
C4—H4B	0.9700	C13—H13B	0.9600
C5—C6	1.537 (5)	C13—H13C	0.9600
C5—H5A	0.9700	C14—H14A	0.9600
C5—H5B	0.9700	C14—H14B	0.9600
C6—C7	1.518 (4)	C14—H14C	0.9600
C6—H6A	0.9700	C15—H15A	0.9600
C6—H6B	0.9700	C15—H15B	0.9600
C7—C14	1.539 (5)	C15—H15C	0.9600
C7—C15	1.545 (5)	C16—H16A	0.9600
C7—C8	1.574 (4)	C16—H16B	0.9600
C8—C9	1.515 (3)	C16—H16C	0.9600
C8—H8	0.9800	N1—H1	0.8600
C9—C10	1.416 (4)		
			
C2—C1—C12	118.0 (2)	N1—C9—C8	113.8 (2)
C2—C1—C8	118.5 (2)	C10—C9—H9	115.3
C12—C1—C8	112.99 (19)	N1—C9—H9	115.3
C2—C1—C3	58.42 (15)	C8—C9—H9	115.3
C12—C1—C3	122.04 (19)	C9—C10—C16	121.3 (3)
C8—C1—C3	116.70 (19)	C9—C10—N1	60.2 (2)
C3—C2—C1	61.83 (16)	C16—C10—N1	109.1 (3)
C3—C2—Cl1	118.46 (19)	C9—C10—C11	121.0 (2)
C1—C2—Cl1	118.6 (2)	C16—C10—C11	116.7 (3)
C3—C2—Cl2	120.6 (2)	N1—C10—C11	110.1 (3)
C1—C2—Cl2	122.04 (18)	C10—C11—C12	113.8 (2)
Cl1—C2—Cl2	108.84 (14)	C10—C11—H11A	108.8
C2—C3—C4	119.4 (2)	C12—C11—H11A	108.8
C2—C3—C13	118.8 (2)	C10—C11—H11B	108.8
C4—C3—C13	112.9 (2)	C12—C11—H11B	108.8
C2—C3—C1	59.75 (15)	H11A—C11—H11B	107.7
C4—C3—C1	116.7 (2)	C1—C12—C11	112.2 (2)
C13—C3—C1	119.6 (2)	C1—C12—H12A	109.2
C3—C4—C5	110.9 (2)	C11—C12—H12A	109.2
C3—C4—H4A	109.5	C1—C12—H12B	109.2
C5—C4—H4A	109.5	C11—C12—H12B	109.2
C3—C4—H4B	109.5	H12A—C12—H12B	107.9
C5—C4—H4B	109.5	C3—C13—H13A	109.5
H4A—C4—H4B	108.0	C3—C13—H13B	109.5
C6—C5—C4	114.9 (2)	H13A—C13—H13B	109.5
C6—C5—H5A	108.5	C3—C13—H13C	109.5
C4—C5—H5A	108.5	H13A—C13—H13C	109.5
C6—C5—H5B	108.5	H13B—C13—H13C	109.5
C4—C5—H5B	108.5	C7—C14—H14A	109.5
H5A—C5—H5B	107.5	C7—C14—H14B	109.5
C7—C6—C5	118.4 (3)	H14A—C14—H14B	109.5
C7—C6—H6A	107.7	C7—C14—H14C	109.5
C5—C6—H6A	107.7	H14A—C14—H14C	109.5
C7—C6—H6B	107.7	H14B—C14—H14C	109.5
C5—C6—H6B	107.7	C7—C15—H15A	109.5
H6A—C6—H6B	107.1	C7—C15—H15B	109.5
C6—C7—C14	112.5 (3)	H15A—C15—H15B	109.5
C6—C7—C15	106.3 (3)	C7—C15—H15C	109.5
C14—C7—C15	107.0 (3)	H15A—C15—H15C	109.5
C6—C7—C8	112.1 (2)	H15B—C15—H15C	109.5
C14—C7—C8	107.0 (3)	C10—C16—H16A	109.5
C15—C7—C8	111.9 (3)	C10—C16—H16B	109.5
C9—C8—C1	109.9 (2)	H16A—C16—H16B	109.5
C9—C8—C7	112.8 (2)	C10—C16—H16C	109.5
C1—C8—C7	114.2 (2)	H16A—C16—H16C	109.5
C9—C8—H8	106.5	H16B—C16—H16C	109.5
C1—C8—H8	106.5	C9—N1—C10	57.3 (2)
C7—C8—H8	106.5	C9—N1—H1	151.3
C10—C9—N1	62.4 (2)	C10—N1—H1	151.3
C10—C9—C8	123.7 (2)		
